# Economic Analysis of Greenhouse Lighting: Light Emitting Diodes vs. High Intensity Discharge Fixtures

**DOI:** 10.1371/journal.pone.0099010

**Published:** 2014-06-06

**Authors:** Jacob A. Nelson, Bruce Bugbee

**Affiliations:** Crop Physiology Laboratory, Department of Plant Soils and Climate, Utah State University, Logan, Utah, United States of America; Mount Allison University, CANADA

## Abstract

Lighting technologies for plant growth are improving rapidly, providing numerous options for supplemental lighting in greenhouses. Here we report the photosynthetic (400–700 nm) photon efficiency and photon distribution pattern of two double-ended HPS fixtures, five mogul-base HPS fixtures, ten LED fixtures, three ceramic metal halide fixtures, and two fluorescent fixtures. The two most efficient LED and the two most efficient double-ended HPS fixtures had nearly identical efficiencies at 1.66 to 1.70 micromoles per joule. These four fixtures represent a dramatic improvement over the 1.02 micromoles per joule efficiency of the mogul-base HPS fixtures that are in common use. The best ceramic metal halide and fluorescent fixtures had efficiencies of 1.46 and 0.95 micromoles per joule, respectively. We also calculated the initial capital cost of fixtures *per photon delivered* and determined that LED fixtures cost five to ten times more than HPS fixtures. The five-year electric plus fixture cost per mole of photons is thus 2.3 times higher for LED fixtures, due to high capital costs. Compared to electric costs, our analysis indicates that the long-term maintenance costs are small for both technologies. If widely spaced benches are a necessary part of a production system, the unique ability of LED fixtures to efficiently focus photons on specific areas can be used to improve the photon capture by plant canopies. Our analysis demonstrates, however, that the cost per photon delivered is higher in these systems, regardless of fixture category. The lowest lighting system costs are realized when an efficient fixture is coupled with effective canopy photon capture.

## Introduction

Rapid advances in lighting technology and fixture efficiency provide an expanding number of options for supplemental lighting in greenhouses, including numerous LED fixtures (light emitting diode, see [1], [2] for a history of LED lighting in horticulture). Significant improvements have been made in all three high intensity discharge (HID, which includes high pressure sodium, HPS, and ceramic metal halide, CMH) fixture components: the lamp (often referred to as the bulb), the luminaire (often referred to as the reflector) and the ballast. High pressure sodium fixtures with electronic ballasts and double-ended lamps are now 1.7 times more efficient than older mogul-base HPS fixtures.

**Figure 1:**
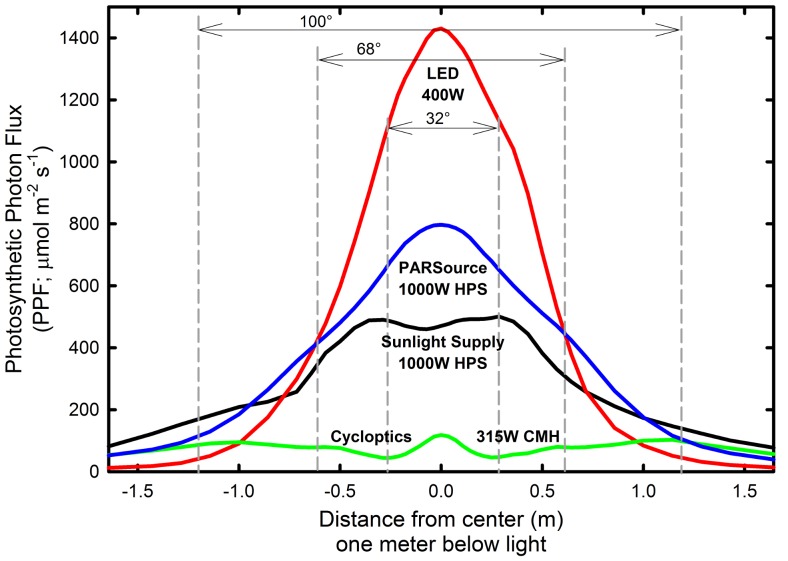
The photon distribution of four fixtures with similar photon efficiency. Each line represents a cross section of the photon intensity below the fixture. The LED fixture (Lighting Sciences Group) uses optics to achieve a narrow distribution, with the majority of the photons falling in a concentrated pattern directly below the fixture. Conversely, the Cycloptics ceramic metal halide fixture is designed for even light distribution, and therefore casts uniform radiation over a large surface area. Since the area increases exponentially as the distance from the center increases, an equal photon flux farther from the center represents a larger quantity of total photons.

**Figure 2:**
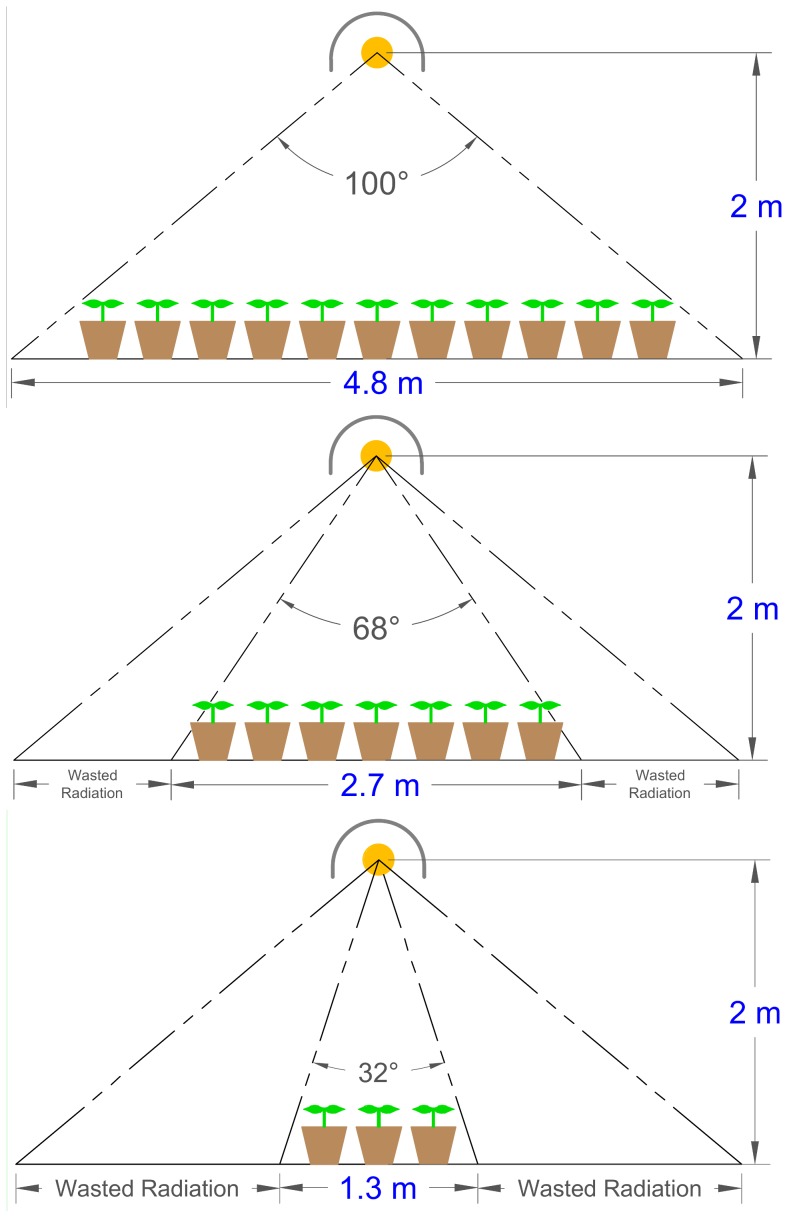
Canopy photon capture efficiency. As the plant growth area under the fixture gets smaller, wasted radiation often increases. This figure illustrates the concept of canopy photon capture efficiency. Two meters was chosen as a typical mounting height, but this can be scaled as a unit-less ratio. Multiple overlapping fixtures are typically used to minimize PPF variation over a large area.

Lighting technologies vary widely in how radiation is distributed (Figure 1). There is no ideal pattern of radiation distribution for every application. In large greenhouses with small aisles and uniformly spaced plants, the broad, even output pattern typically emitted from HPS fixtures provides uniform (little variation over a large area) light distribution and increased capture of photosynthetic photons. In smaller greenhouses with spaced benches, the more focused pattern typically found in LED fixtures can maximize radiation transfer to plant leaves. As the area (height of width) covered by plants increases, the need for more focused radiation decreases (Figure 2).

In greenhouse applications, selection among lighting options should primarily be made based on the cost to deliver photons to the plant canopy surface. This analysis includes two parameters: 1) the fundamental fixture efficiency, measured as micromoles of photosynthetic photons per joule of energy input, and 2) the canopy photosynthetic (400–700 nm) photon flux (PPF) capture efficiency, which is the fraction of photons transferred to the plant leaves.

### Electrical efficiency for plant growth is best measured as µmoles per Joule

The electrical efficiency of lamps is often expressed using units for human light perception (efficacy; lumens or foot-candles out per watt in) or energy efficiency (radiant watts out per electrical watt in). Photosynthesis and plant growth, however, is determined by moles of photons. It is thus important to compare lighting efficiency based on photon efficiency, with units of micromoles of photosynthetic photons per joule of energy input. This is especially important with LEDs where the most electrically efficient colors are in the deep red and blue wavelengths. A dramatic example of this is the comparison of red, blue, and cool white LEDs (Table 1). The lower radiant energy content of red photons allows more photons to be delivered per unit of input energy (radiant energy is inversely proportional to wavelength, Planck's Equation). Conversely, blue LEDs can have a 53% higher energy efficiency (49% vs. 32%) but only a 9% higher photon efficiency (1.87 vs. 1.72).

**Table 1:** Efficiency of individual LEDs at a drive current of 700

LED Color	Peak wavelength or color temperature	Photon efficiency^z^ (µmol/J)	Electrical efficiency^y^ (%)	Luminous efficiency^x^ (lm/W)
Cool white	5650 Kelvin	1.52	33	111
Red	655 nm	1.72	32	47
Blue	455 nm	1.87	49	17

z -Photon efficiency is the most appropriate measure for photosynthesis.

y -The relationship between electrical efficiency and photon efficiency is dependent on wavelength (Plank's equation E = hc/λ).

x -Luminous efficiency is shown to demonstrate how inappropriate it is as an indicator of lighting efficiency for plants.

### Effect of light quality

**Figure 3:**
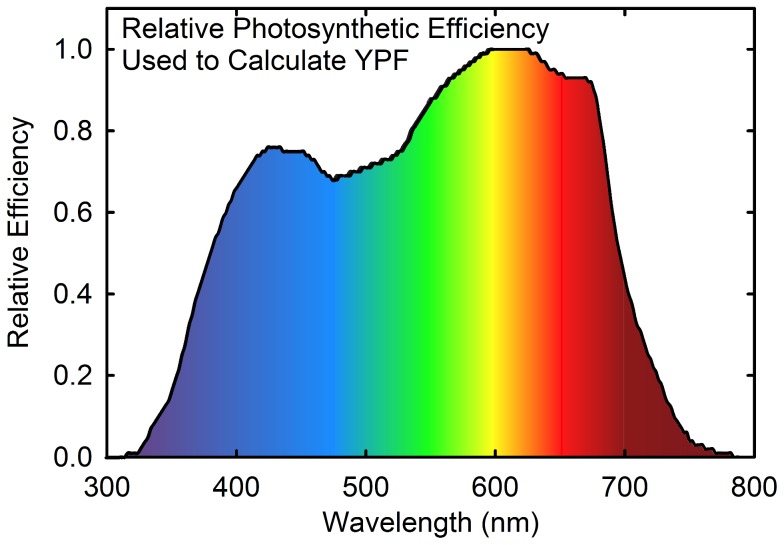
Yield photon flux curve. Effect of wavelength on relative photosynthesis per incident photon for a single leaf in low light (less than 150 µmol m^−2^ s^−1^) [4].

There is considerable misunderstanding over the effect of light quality on plant growth. Many manufacturers claim significantly increased plant growth due to light quality (spectral distribution or the ratio of the colors). A widely used estimate of the effect of light quality on photosynthesis comes from the Yield Photon Flux (YPF) curve, which indicates that orange and red photons between 600 to 630 nm can result in 20 to 30% more photosynthesis than blue or cyan photons between 400 and 540 nm (Figure 3)[3], [4]. When light quality is analyzed based on the YPF curve, HPS lamps are equal to or better than the best LED fixtures because they have a high photon output near 600 nm and a low output of blue, cyan, and green light [5].

The YPF curve, however, was developed from short-term measurements made on single leaves in low light. Over the past 30 years, numerous longer-term studies with whole plants in higher light indicate that light *quality* has a much smaller effect on plant growth rate than light *quantity* [6], [7]. Light quality, especially the fraction of blue light, has been shown to alter cell expansion rate, leaf expansion rate[8], plant height and plant shape in several species [9]–[11], but it has only a small direct effect on photosynthesis. The effects of light quality on fresh or dry mass in whole plants typically occur under low or no sunlight conditions, and are caused by changes in leaf expansion and radiation capture during early growth [6].

### Unique aspects of LED fixtures

**Figure 4:**
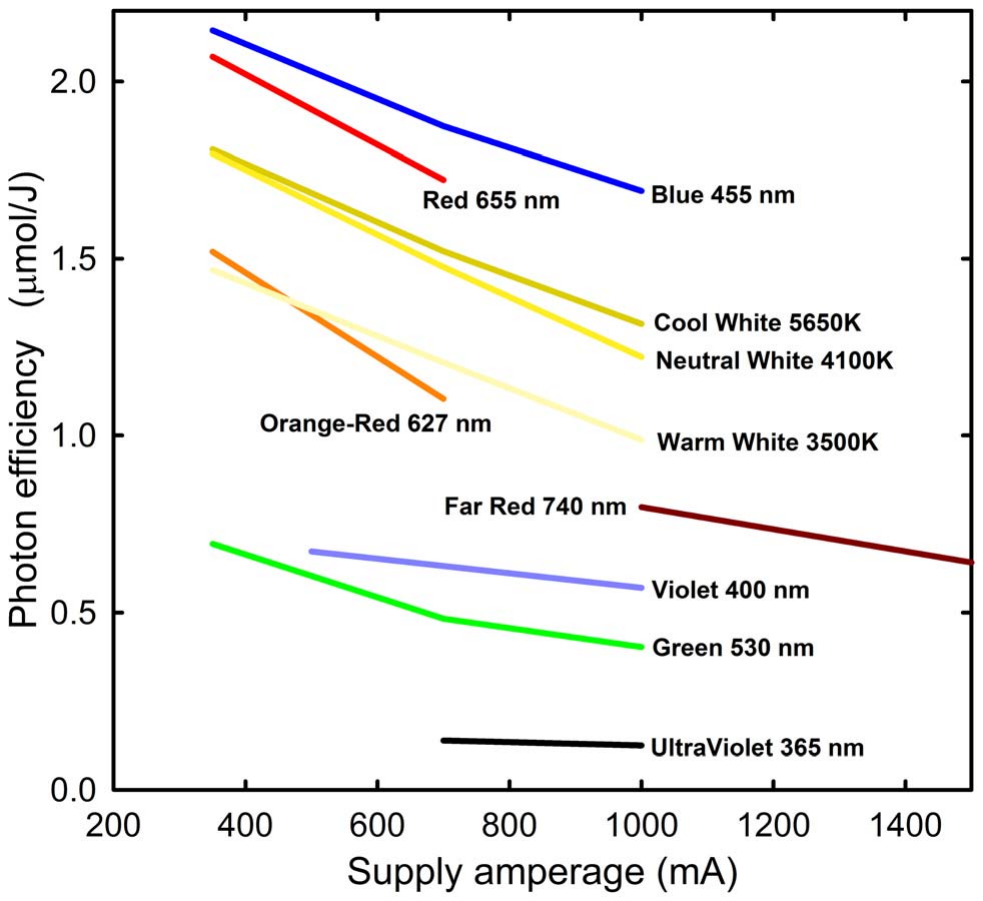
Effect of drive amperage and color on photon efficiency of LEDs. Data for Philips Lumileds LEDs (May 2014), courtesy of Mike Bourget, Orbitec.

The most electrically efficient colors of LEDs, based on moles of photosynthetic photons per joule, are blue, red, and cool white, respectively (Figure 4), so LED fixtures generally come in combinations of these colors. LEDs of other colors can be used to dose specific wavelengths of light to control aspects of plant growth [12], due to their monochromatic nature (see [13] for a review of unique LED applications). Ultraviolet (UV) radiation is typically absent in LED fixtures because UV LEDs significantly reduce fixture efficiency. Sunlight has 9% UV (percent of PPF), and standard electric lights have 0.3 to 8% UV radiation (percent of PPF)[5]. A lack of UV causes disorders in some plant species (e.g. Intumescence; [14]) and this is a concern with LED fixtures when used without sunlight. LED fixtures for supplemental photosynthetic lighting also have minimal far-red radiation (710 to 740 nm), which decreases the time to flowering in several photoperiodic species [15]. Green light (530 to 580 nm) is low or absent in most LED fixtures and these wavelengths better penetrate through the canopy and are more effectively transmitted to lower plant leaves [16]. The lack of UV, green, and far-red wavelengths, however, should be minimal when LEDs are used in greenhouses, because most of the radiation comes from broad spectrum sunlight.

Our objective is to help growers and researchers select the most cost effective fixture options for supplemental lighting in greenhouses. To achieve this goal we measured two fundamental components of each fixture: 1) the efficiency of conversion of electricity to photosynthetic photons that are delivered to a horizontal surface below the lamp, and 2) the distribution pattern of these photons below the fixture.

## Materials and Methods

### Fixture efficiency

Measurements of fixture efficiency (lamp, luminaire, and ballast) were made by integrating sphere and flat-plane integration techniques. The integrating sphere measurements were made by a certified testing laboratory (TÜV SÜD America) that specializes in the measurement of the efficiency of lighting fixtures using the IES LM79-08 measurement standard [17]. Radiometric output was converted to photon output at each nanometer interval using Plank's Equation and then integrated from 400 to 700 nanometers. The radiation measurements were calibrated to NIST reference standards. These measurements of fixture efficiency are considered repeatable to within 1%.

### Flat plane integration

Measurements were made in a dark room with flat black walls using a quantum sensor (LI-COR model LI-190, Lincoln, NE, USA), that was calibrated for each fixture with an NIST-traceable calibrated spectroradiometer (model PS-200, Apogee Instruments, Logan, UT, USA). This calibration is necessary to correct for small spectral errors (±3%) in the quantum sensor that occur because of imperfect matching of the ideal quantum response [18]. Measurements were made in three radial, straight lines below a level fixture and spatially integrated over a flat plane below the fixture to determine total photon output. Measurements were made 2.5 cm apart near the center, increasing to 10 cm near the perimeter as PPF variation decreased (121 measurements total). Fixtures were mounted 0.7 meters above the surface and measurements were made up to a 1.5 meter radius from the center and extrapolated to infinity using an exponential decay function. Fixture height is optional, depending on the size of the room and measurement area as long as measurement resolution captures the spatial variation in fixture output. The flat-plane integration measurements were used to quantify the pattern of photon distribution from the fixture. Total fixture output from these measurements was similar to measurements made using an integrating sphere (Table 2). When redundant measurements were available, the integrating sphere measurements were used to quantify fixture efficiency. Power draw and electrical characteristics were measured using a multimeter and a current clamp (Fluke model 289, Everett, WA, USA).

**Table 2:** Efficiency of fixtures using integrating sphere measurements compared with flat-plane integration.

	TÜV SÜD America integrating sphere			USU^z^ flat plane integration			flat plane/integrating sphere^y^
Fixture	Elec. input (W or J/s)	Photon output (µmol/s)	Photon efficiency (µmol/J)^x^	Elec. input (W or J/s)	Photon output (µmol/s)	Photon efficiency (µmol/J)^x^	(µmol/J)/(µmol/J)
Gavita Pro 1000DE	1033	1751	1.70	1041	1814	1.74	2.7%
ePapillion 1000W	1041	1767	1.70	1037	1937	1.87	9.1%
LSG violet	384	653	1.70	391	628	1.61	−6.0%
SPYDR 600	326	541	1.66	332	575	1.73	4.4%
LSG red/white	390	634	1.63	397	601	1.51	−7.5%
Illumitex NeoSol	279	390	1.40	281	386	1.38	−1.8%
ParSource GLXII	1026	1334	1.30	1008	1433	1.42	8.6%
Lumigrow Pro 325	304	390	1.29	304	355	1.17	−10.1%
California Light Works SOLARSTORM	337	350	1.04	343	331	0.96	−7.7%
Black Dog BD360U	339	339	1.00	346	323	0.93	−7.2%
Apache AT120WR	169	163	0.96	167	150	0.90	−7.2%
iGrow 400W	394	374	0.95	397	354	0.89	−6.5%
Lumigrow es330	318	284	0.90	317	270	0.85	−5.1%
Hydrogrow Sol 9	423	378	0.89	430	396	0.92	2.9%

z -Utah State University

y -The flat-plane integration may have made an inadequate number of measurements to fully characterize the output of some of the lamps. The electric consumption (watts) by the fixture was nearly identical among test sites and did not likely have a significant effect of efficiency.

x - Photon Output per Electrical Input (µmol per second divided by joules per second).

### Cost of electricity

In the United States, commercial electric rates vary widely by region, ranging from $0.07 in Idaho to $0.17 in New York, with residential rates averaging $0.02 higher, and industrial rates $0.02 lower. Electric rates in Europe, and many other countries, can be more than double the rates in the United States. As electricity becomes more expensive, improved lighting becomes more valuable. See U.S. Energy Information Administration for a summary of current electric rates by state and region (accessed April 2014). We used a discounted cash flow model assuming a 5% per year cost of capital on future electrical costs.

## Results

**Figure 5:**
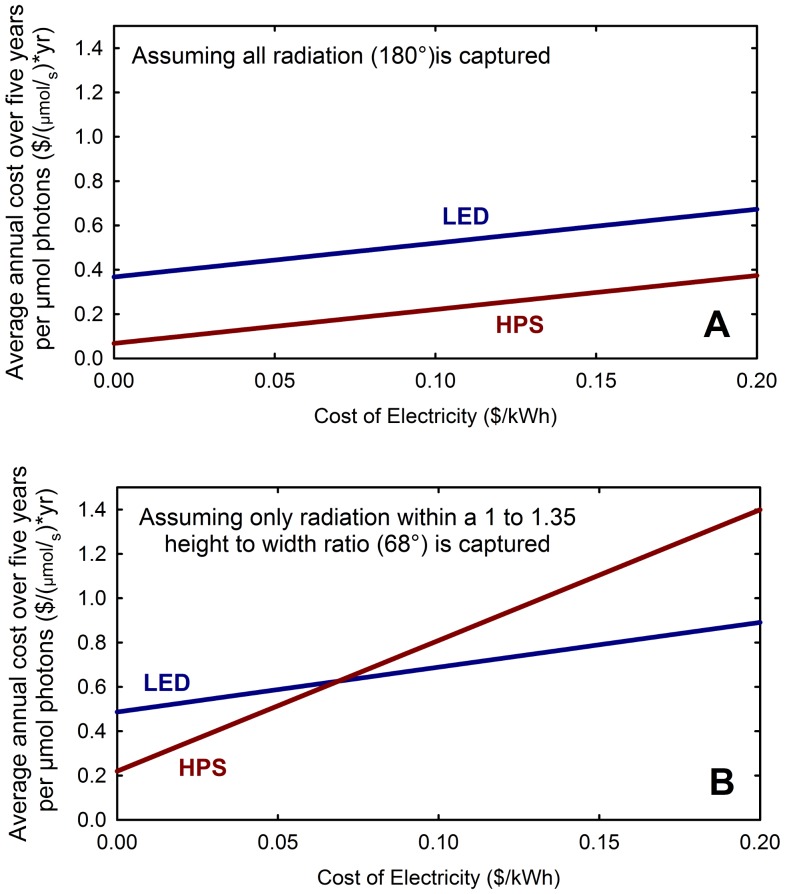
Effect of electricity price on average annual cost over five years for two capture scenarios. (A) When all radiation is assumed captured, the most efficient HPS fixture (Gavita) has a lower average annual five-year cost per photon than the most efficient LED fixture (Red/Blue fixture, Lighting Sciences Group). (B) When only a narrow region below the fixture (68°) is considered to be captured (e.g. on benches), the LEDs can have a lower cost per photon then HPS fixtures, but the cost per photon increases for both fixtures.

The photon efficiency (micromoles per joule) and cost per mole of photons for four categories of lighting technologies (HPS, LED, ceramic metal halide, and fluorescent), in 22 fixtures, are shown in Table 3. One fixture of each model was tested. This table also shows the five-year electric plus fixture costs per mole of photons. Most fixtures (lamp, luminaire and ballast) are now more efficient than the common 1000-W magnetic-ballast, mogul-base HPS fixtures (i.e. Sunlight Supply, 1.02 µmol per joule). If photons coming out of the fixture at all downward angles are considered (180°), the capital cost of the most efficient 400-W LED fixtures we tested is five to seven times more per photon than the 1000-W, double-ended, electronic ballast HPS fixtures (Gavita, ePapillion, Table 3). The high capital cost of LEDs makes the five year cost per mole of photons more than twice that of HPS fixtures (Table 3 and Figure 5A).

**Table 3:** Photon efficiency and cost per mole of photons, assuming all photons (180°) are captured by plants.

Lamp type and Ballast	Fixture producer^z^	Electrical input (J/s or watts)	Photon output^y^ (µmol/s)	Photon efficiency^x^ (µmol/J)	Cost of one fixture^w^ ($)	Fixtures needed per millimol/s^v^	Fixture cost per mol/s $/(mol/s)	Electric cost per µmol photons^u^ $/(µmol/s)yr	Five year electric cost per µmol photons^t^ $/(µmol/s)yr
**High Pressure Sodium**									
400 W magnetic	Sunlight Supply	443	416	0.94	$200	2.40	$0.48	$0.35	$0.40
1000 W magnetic	Sunlight Supply	1067	1090	1.02	$275	0.92	$0.25	$0.32	$0.33
1000 W magnetic	PARsource GLXI	1004	1161	1.16	$350	0.86	$0.30	$0.29	$0.31
1000 W electronic	PARsource GLXI	1024	1333	1.30	$380	0.75	$0.29	$0.25	$0.28
1000 W electronic	PARsource GLXII	1026	1334	1.30	$310	0.75	$0.23	$0.25	$0.27
1000 W electronic	Gavita	1033	1751	1.70	$500	0.57	$0.29	$0.19	$0.23
1000 W electronic	ePapillon	1041	1767	1.70	$600	0.57	$0.34	$0.19	$0.24
**LED**									
red/blue	LSG	384	653	1.70	$1,200	1.53	$1.84	$0.19	$0.54
red/white	BML	326	541	1.66	$1,000	1.85	$1.85	$0.20	$0.54
red/white	LSG	390	634	1.63	$1,200	1.58	$1.89	$0.20	$0.55
red/white	Illumitex	279	390	1.40	$1,400	2.56	$3.59	$0.24	$0.92
red/white/blue	Lumigrow (Pro 325)	304	390	1.29	$1,000	2.56	$2.56	$0.26	$0.73
red/white	California Lightworks	337	350	1.04	$1,000	2.85	$2.85	$0.32	$0.85
multiple	Black Dog	339	339	1.00	$950	2.95	$2.80	$0.33	$0.85
red/white	Apache	169	163	0.96	$860	6.14	$5.28	$0.34	$1.35
red/blue	Lumigrow (ES330)	318	284	0.90	$1,200	3.52	$4.22	$0.37	$1.16
red/white	Hydrogrow	423	378	0.89	$1,300	2.64	$3.44	$0.37	$1.01
**Ceramic Metal Halide**									
315 W 3100 K	Cycloptics	337	491	1.46	$640	2.04	$1.30	$0.23	$0.46
315 W 4200 K	Cycloptics	340	468	1.38	$640	2.14	$1.37	$0.24	$0.48
2@315 W 3100 K	Boulderlamp	651	817	1.25	$1,000	1.22	$1.22	$0.26	$0.47
**Fluorescent**									
400 W induction	iGrow	394	374	0.95	$1,200	2.68	$3.21	$0.35	$0.94
60 W	T8	58	48	0.84	$40	20.77	$0.83	$0.40	$0.51

z - See Table S1 for a list of fixture manufacturers and model numbers.

y - Integrated total photon output of fixture.

x - Photon Output per Electrical Input (µmol per second divided by joules per second).

w -Cost of fixtures as of April 2014.

v -The number of fixtures to get a total photon output of one millimol (1000 µmol) of photons per second.

u -Assumes 3000 hours per year operation and $0.11/kWh.

t -Cost of fixture (multiplied by fixtures needed) plus cost of electricity over 5 years. We used a discounted cash flow model assuming a 5% per year cost of capital. Installation and maintenance costs were assumed to be similar for all lamp types and were not included in this calculation.

**Figure 6:**
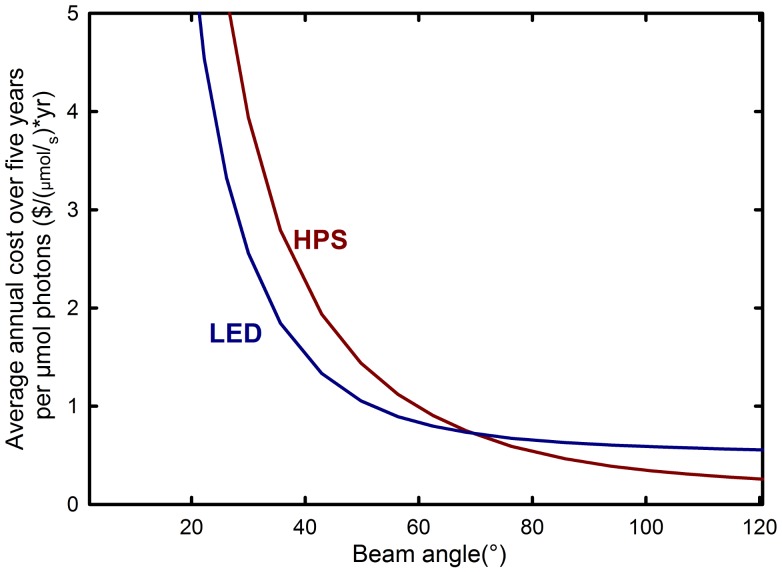
Effect of canopy capture efficiency on average annual cost over five years. The cost per mole of photons for LEDs (Red/Blue LED from Lighting Sciences Group) becomes more favorable than the best HPS fixtures (Gavita) when the lighting area is less than 68° from center, assuming $0.11 per kWh cost of electricity and 3000 hours per year use (approximate cumulative operation time at latitudes from 40 to 50 degrees).

Table 3 assumes that all of the photons emitted from the fixture are absorbed by plant leaves. In Table 4, the area under the fixture in which the photons are considered captured by plants is progressively reduced, and the cost per mole of photons increases as more photons are lost around the perimeter. When only highly focused radiation is considered useful (34°), some LED fixtures have a lower cost per photon than the best HPS fixtures (Table 4, Figure 1, Figure 5B and Figure 6), but because photons are lost around the perimeter at this narrow angle, the cost per photon absorbed by plants is much greater. The lowest cost per photon is realized when a large canopy can be arranged to capture the photons.

**Table 4:** Cost per mole photons for four capture assumptions.

		Assuming all radiation (180°) is captured		Assuming radiation within a 1 to 2.38 height to width ratio (100°) is captured		Assuming radiation within a 1 to 1.35 height to width ratio (68°) is captured	
Lamp type and Ballast	Fixture producer^z^	Fixtures needed per mmol/s^y^	Five year electric cost per µmol photons^x^ $/(µmol/s)yr	Fixtures needed per mmol/s^y^	Five year electric cost per µmol photons^x^ $/(µmol/s)yr	Fixtures needed per mmol/s^y^	Five year electric cost per µmol photons^x^ $/(µmol/s)yr
**High Pressure Sodium**							
400 W magnetic	Sunlight Supply	2.40	$0.40	3.99	$0.66	8.51	$1.42
1000 W magnetic	Sunlight Supply	0.92	$0.33	1.71	$0.61	3.60	$1.30
1000 W magnetic	PARsource GLXI	0.86	$0.31	1.31	$0.47	2.82	$1.01
1000 W electronic	PARsource GLXI	0.75	$0.28	1.14	$0.42	2.49	$0.92
1000 W electronic	PARsource GLXII	0.75	$0.27	1.33	$0.47	2.81	$1.00
1000 W electronic	Gavita	0.57	$0.23	0.96	$0.38	2.12	$0.84
1000 W electronic	ePapillon	0.57	$0.24	1.46	$0.61	3.47	$1.45
**LED**							
red/blue	LSG	1.53	$0.54	1.62	$0.57	2.03	$0.71
red/white	BML	1.85	$0.54	2.13	$0.62	3.17	$0.93
red/white	LSG	1.58	$0.55	1.67	$0.59	2.09	$0.73
red/white	Illumitex	2.56	$0.92	2.66	$0.96	3.82	$1.37
red/white/blue	Lumigrow (Pro 325)	2.56	$0.73	3.05	$0.87	4.95	$1.42
red/white	California Lightworks	2.85	$0.85	3.09	$0.92	4.92	$1.46
multiple	Black Dog	2.95	$0.85	4.43	$1.27	8.64	$2.48
red/white	Apache	6.14	$1.35	6.58	$1.45	8.21	$1.81
red/blue	Lumigrow (ES330)	2.64	$1.01	2.82	$1.07	4.33	$1.65
red/white	Hydrogrow	3.52	$1.16	5.05	$1.67	10.70	$3.54
**Ceramic Metal Halide**							
315 W 3100 K	Cycloptics	2.04	$0.46	5.43	$1.22	19.55	$4.38
315 W 4200 K	Cycloptics	2.14	$0.48	5.72	$1.29	20.71	$4.66
2@315 W 3100 K	Boulderlamp	1.22	$0.47	1.56	$0.60	2.90	$1.12
**Fluorescent**							
400 W induction	iGrow	2.68	$0.94	4.69	$1.65	10.17	$3.58
60 W	T8	20.77	$0.51	38.03	$0.93	83.81	$2.05

z - See Table S1 for a list of fixture manufacturers and model numbers.

y -The number of fixtures to get 1 millimol (1000 µmol) of photons per second.

x -Cost of fixture (multiplied by fixtures needed) plus cost of electricity over 5 years. We used a discounted cash flow model assuming a 5% per year cost of capital. Installation and maintenance costs were assumed to be similar for all lamp types and were not included in this calculation.

## Discussion

### Importance of photon capture

As reviewed in the introduction, lighting system efficiency is the combined effect of efficient fixtures and efficient canopy photon capture efficiency. Precision luminaires, lenses (e.g. model vivid white, Lighting Sciences Group inc.), or adjustable angle LEDs (e.g. model SPYDR 600, BML inc.) can be used to apply highly focused lighting specifically to the plant growth areas. This is valuable in small greenhouses with widely spaced benches. Canopy photon capture efficiency can be maximized, to above 90%, for large greenhouses with narrow aisles regardless of fixture type. The use of LED intracanopy lighting can increase capture rates to near 100%, and may have other beneficial effects such as increased light sharing with intracanopy leaves [19], [20]. The concentration of heat from HID fixtures makes intracanopy lighting infeasible with high wattage HPS fixtures. Just as precision irrigation can improve water efficiency, precision lighting can improve electrical efficiency.

### Effect of fixture shadow

All fixtures block radiation from the sun, and the shadow is proportional to the size of the fixture. For the same photon output, 400-W HPS, ceramic metal halide, fluorescent, and LED fixtures block significantly more sunlight than 1000-W HPS fixtures. We did not include the effect of the shadow in this analysis, but this effect significantly favors the more energy dense, higher wattage HPS fixtures. In the long-term, LEDs can take advantage of innovative design options like mounting along greenhouse support structures, which could provide light without extra shading. Longer, narrower LED fixtures may be preferable to rectangular fixtures because the duration of the shadow is shorter. Fluorescent fixtures, including induction fluorescent, have large shadows relative to their photon output (and have low photon efficiencies) and are therefore generally not economical for greenhouse lighting.

### Installation, annual maintenance costs, and life expectancy

Installation costs include wiring for fixtures and physically hanging the fixture. In our experience, the cost of installation is similar for both fixture types, although installation costs can be reduced by fewer, higher wattage fixtures. The annual maintenance costs are small relative to the cost of the electricity, and these costs are better established for HPS fixtures than for LED fixtures. Maintenance costs are largely determined by the life expectancy of the fixture.

Double-ended HPS lamps (1000-W) have a life expectancy of 10,000 hours to 90% survival (based on manufacturer literature), or 3.3 years when used an average of 8 hours per day or 3,000 hours per year (traditional mogul-base lamps have industry reported life expectancies of 10,000 to 17,000 hours, to 90% survival, and cost approximately $40). The cost of a 1000-W, double-ended replacement lamp is about $140, which averages to $28 per year if we assume a lamp will be replaced once in the first five years. This lamp replacement cost can increase to $30 to $35 per year when the labor to replace the bulb is included, but this is a small amount compared to the approximately $600 per year annual electric cost to operate the fixture. Adding the cost of lamp replacement increases the five-year cost of operation by approximately 5%.

When operated at favorable temperatures, individual LEDs generally have a predicted lifetime (to 70% of the initial light output) of up to 50,000 hours, about 16.7 years when used an average 8 hours per day or 3000 hours per year. The economic life for LED fixtures for plant lighting has not been established, but it depends on the value of the product being produced. The high capital cost of replacement means that LED fixtures would be operated longer, in spite of diminished photon output. Replacement of individual LEDs is more expensive than replacing an HID lamp. The life expectancy of LEDs is reduced if they are driven by higher amperage to achieve a higher output, or exposed to high temperatures. Fixtures may be warmed by radiation from sunlight. The cooler the LED temperature, the longer they last. Power supplies, fans, and other components in LED fixtures can fail well before the LEDs themselves. Fan failure would increase LED temperature and may not be immediately noticed by the user. These components are replaceable, but the labor costs to change fixture components increases operating costs.

For these reasons we have not included a differential operating cost between LED and HPS fixtures. We assumed that maintenance costs will be minimal during the first five years for all types of fixtures. Electronic ballasts for 1000-W HPS lamps are still a relatively new technology, and fixtures vary in quality. We have experienced premature failures of LED power supplies, LED circuit boards, HPS lamps, and electronic HPS ballasts in our greenhouse operations. LED fixtures with improved power supplies and optimized operating amperages are available from reputable manufacturers. Improvements in these new technologies are occurring rapidly.

### Importance of PPF uniformity

PPF uniformity is critical in many greenhouse applications, especially in floriculture. It is easier to achieve uniformity with fixtures that have broad distribution of photons. Economically, the value of uniform plants may outweigh the cost of wasted photons. Uniformity has been well characterized and modeled with HID lights [21], [22], but these techniques have not yet been rigorously applied to LED fixtures. Ciolkosz et al. [23] showed that uniform light on the perimeter of a greenhouse requires higher fixture densities in the outer rows, and consequentially may increase the amount of radiation lost beyond the edge of the growing area, decreasing canopy photon capture. HPS fixtures with narrower focus luminaires tend to have lower photon efficiencies.

### Effect of fixture efficiency on heating and cooling costs

Improved electrical efficiency reduces the cooling load in a greenhouse, which increases the value of efficient fixtures when cooling is required. The best HPS and LED fixtures have nearly identical efficiency, so cooling costs are similar for both fixture categories. The ability to cycle LED fixtures, which prematurely ages other fixture types, could be used to stabilize the heating and cooling load in a greenhouse during partly cloudy days, which could improve temperature control and increase the lifetime of cooling system equipment.

Additional thermal radiation is useful in warming the plant canopy during the heating season, but is detrimental if the canopy is too warm. When sunlight supplies adequate PPF, supplemental lighting is usually turned off.

### Conclusions

The most efficient HPS and LED fixtures have equal efficiencies, but the initial capital cost per photon delivered from LED fixtures is five to ten times higher than HPS fixtures. The high capital cost means that the five-year cost of LED fixtures is more than double that of HPS fixtures. If widely spaced benches are a necessary part of a production system, LED fixtures can provide precision delivery of photons and our data indicate that they can be a more cost effective option for supplemental greenhouse lighting.

Manufacturers are working to improve all types of lighting technologies and the cost per photon will likely continue to decrease as new technologies, reduced prices, and improved reliability become available.

## Supporting Information

Table S1 **Fixture manufacturer and model numbers.** A table containing the mixture manufactuere and model numbers of all fixtures referenced in this study.
(PDF)
