# Supplementary material for: Economic Analysis of Greenhouse Lighting: Light Emitting Diodes vs. High Intensity Discharge Fixtures
Source: PLoS One. 2014 Jun 6;9(6):e99010. doi: 10.1371/journal.pone.0099010 (PMC4048233; doi:10.1371/journal.pone.0099010)
Supplement: Table S1 — Fixture manufacturer and model numbers. A table containing the mixture manufactuere and model numbers of all fixtures referenced in this study. (PDF) [file pone.0099010.s001.pdf]

Table S1. Fixture manufacturer and model numbers.

Lamp type and Ballast	Fixture producer	Model number
**High Pressure Sodium**		
400 W magnetic	Sunlight Supply	Sunstar
1000 W magnetic	Sunlight Supply	Sunstar
1000 W magnetic	PARsource GLXI	GLX I
1000 W electronic	PARsource GLXI	GLX I
1000 W electronic	PARsource GLXII	GLX II
1000 W electronic	Gavita	GAN Electronic 1000W
1000 W electronic	ePapillon	ePapillon 1000W
**LED**		
390 W red/ blue	LSG (Lighting Sciences Group)	Violet
333 W red/ white	BML	SPYDR 600
390 W red / white	LSG (Lighting Sciences Group)	Vivid White
300 W red/ white	Illumitex	NeoSol NS
325 W red/ white/ blue	Lumigrow (Pro 325)	Pro 325
350 W red/ white	California Lightworks	SolarStorm 440
340 W multiple	Black Dog	BD360-U
120 W red/ white	Apache	AT120WR
330 W red/ blue	Lumigrow (ES330)[z]	ES 330
450 W red/ white	Hydrogrow	Sol 9
**Ceramic Metal Halide**		
315 W 3100 K	Cycloptics	All-Bright
315 W 4200 K	Cycloptics	All-Bright w/ 4200k lamp
2@315 W 3100 K	Boulderlamp	Sun-Bright 630W
**Fluorescent**		
400 W induction	iGrow	IGF-400W
60 W T8 tubes	General Electric	F32T8 SP41 ECO

[z]-The Lumigrow ES330 was discontinued in 2013.
